# Remission of Statin-associated Autoimmune Necrotizing Myopathy With Sequential Immunotherapy: A Well-studied Patient

**DOI:** 10.1210/jcemcr/luaf037

**Published:** 2025-03-27

**Authors:** Francisco J Gómez-Pérez, Erwin Chiquete-Anaya, Andrea G Moncayo-Sánchez, Armando Gamboa-Domínguez, Carlos A Aguilar-Salinas, Roopa Mehta

**Affiliations:** Department of Endocrinology and Lipid Metabolism, Instituto Nacional de Ciencias Médicas y Nutrición Salvador Zubirán, 14080 Mexico City, Mexico; Department of Neurology, Instituto Nacional de Ciencias Médicas y Nutrición Salvador Zubirán, 14080 Mexico City, Mexico; Department of Endocrinology and Lipid Metabolism, Instituto Nacional de Ciencias Médicas y Nutrición Salvador Zubirán, 14080 Mexico City, Mexico; Department of Pathology, Instituto Nacional de Ciencias Médicas y Nutrición Salvador Zubirán, 14080 Mexico City, Mexico; Department of Endocrinology and Lipid Metabolism, Instituto Nacional de Ciencias Médicas y Nutrición Salvador Zubirán, 14080 Mexico City, Mexico; Research direction, Instituto Nacional de Ciencias Médicas y Nutrición Salvador Zubirán, 14080 Mexico City, Mexico; Research Unit for the Study of Metabolic diseases, Instituto Nacional de Ciencias Médicas y Nutrición Salvador Zubirán, 14080 Mexico City, Mexico; Department of Endocrinology and Lipid Metabolism, Instituto Nacional de Ciencias Médicas y Nutrición Salvador Zubirán, 14080 Mexico City, Mexico; Research Unit for the Study of Metabolic diseases, Instituto Nacional de Ciencias Médicas y Nutrición Salvador Zubirán, 14080 Mexico City, Mexico

**Keywords:** necrotizing myopathy, statins, autoimmunity, immunoglobulin therapy

## Abstract

Statins are a group of 3-hydroxy-3-methylglutaryl coenzyme A reductase inhibitors aimed at reducing cholesterol synthesis. Since their discovery in 1970, their use has exponentially increased, becoming a regular preventive treatment for cardiovascular diseases. Among their most common adverse effects are myopathies, with the most severe being autoimmune necrotizing myopathy. We present a case of a woman in her 70s, with high cardiovascular-risk comorbid conditions, including autoimmune hypothyroidism, type 2 diabetes, arterial hypertension, and dyslipidemia. After 5 years on atorvastatin, she developed proximal limb weakness and elevated creatine kinase and creatine kinase-MB levels. A muscle biopsy showed autoimmune necrotizing myopathy and blood work confirmed positive 3-hydroxy-3-methylglutaryl coenzyme A reductase antibodies. Based on these findings, a diagnosis of statin-associated autoimmune necrotizing myopathy was made. In this case, treatment with intravenous immunoglobulin and rituximab resulted in complete remission.

## Introduction

Statins are inhibitors of the enzyme 3-hydroxy-3-methylglutaryl coenzyme A (HMG-CoA) reductase. They are used to lower cholesterol levels and have proven benefit in reducing the risk for cardiovascular diseases [1, 2]. Although statins are generally well tolerated, approximately 2 to 3 of every 100 000 patients receiving these drugs may develop immune-mediated necrotizing myopathy (IMNM) [3]. This is characterized by proximal muscle weakness, elevated levels of muscle enzymes such as creatine kinase (CK) and its muscle isoform (CK-MB), histological evidence of muscle necrosis and degeneration, and circulating anti-HMG-CoA antibodies [3-6]. Discontinuation of the drug is essential on diagnosis; however, symptoms usually persist and progress [7]. Treatments include steroids, intravenous immunoglobulin, and immunosuppressive therapies [8]. We present the case of a patient who developed IMNM after prolonged statin use. Following diagnosis and therapeutic management, the patient experienced complete disease regression.

## Case Presentation

The patient is a 71-year-old woman, with a family history of type 1 diabetes, Hashimoto thyroiditis, and Graves’ disease. Her medical history included Hashimoto thyroiditis with positive anti-thyroglobulin and anti-peroxidase antibodies, type 2 diabetes, arterial hypertension, hepatic steatosis, and mixed hyperlipidemia. She was treated with bezafibrate 200 mg and atorvastatin 20 mg 4 days a week.

After 5 years on atorvastatin, the patient reported difficulty getting up after bending down to pick up her mobile phone. A month later, she developed more pronounced muscle weakness, struggling to climb stairs and comb her hair. Her CK and CK-MB levels were 5674 U/L (94.76 μkat/L) (normal reference range: 30-223 U/L; 0.5-3.72 μkat/L) and 268 ng/mL (268 μg/L) (normal reference range: 0.5-5 ng/mL; 0.5-5 μg/L), respectively. Statin treatment was discontinued. A month later, muscle enzyme concentrations had risen further, with CK reported as 7749 U/L (129.41 μkat/L) (reference range: 30-223 U/L; 0.5-3.72 μkat/L) and CK-MB as 370 ng/mL (370 μg/L) (reference range: 0.5-5 ng/mL; 0.5-5 μg/L). Aspartate aminotransferase (AST) and alanine aminotransferase (ALT) levels were also elevated, with ALT at 384 U/L (6.41 μkat/L) (reference range: 7-52 U/L; 0.12-0.87 μkat/L) and AST at 194 U/L (3.24 μkat/L) (reference range: 13-39 U/L; 0.22-0.65). In the context of the patient, these elevated concentrations were considered to be of muscle origin.

## Diagnostic Assessment

**Figure 1.**
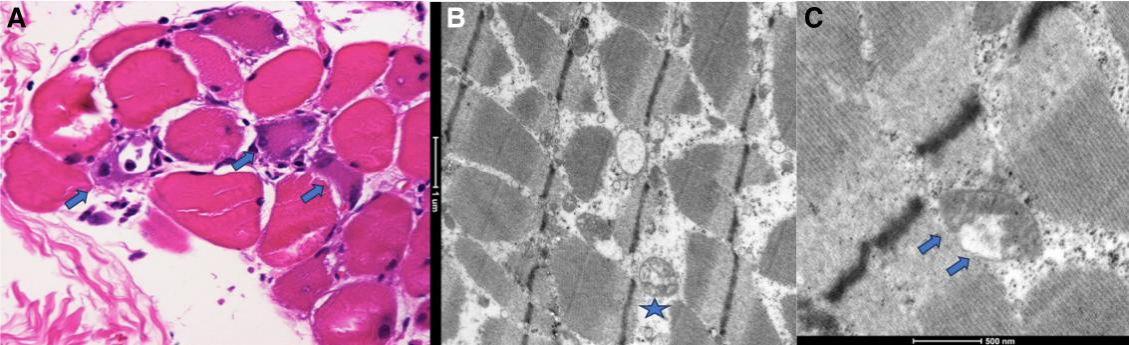
Biopsy image and histopathological report. Cross-section of muscle fibers. Basophilic myofibrils rimmed by lymphocytes and macrophages (arrows) contrast with eosinophilic ones with small peripheral nuclei (A). Ultrastructure of sarcomeres showing Z-line irregularities and enlarged mitochondria (star in B). Crest loss and electron lucent changes were widely present in the mitochondrial internal matrix (arrowhead in c).

Autoantibodies against HMG-CoA reductase were measured at Mayo Clinic Laboratories, yielding a positive result of 160 control units, where values below 20 control units are considered negative. The test was conducted using chemiluminescence immunoassay, a method with a sensitivity of 92.3% and a specificity of 100% [9]. A biopsy of the left deltoid muscle was carried out, which revealed necrotic fibers surrounded by CD4 and CD8 T-lymphocytes mixed with abundant macrophages. Electron microscopy showed a reticular pattern of myofibrils with enlarged mitochondria with crest losses and electron lucent changes, interpreted as part of the process of muscle cellular apoptosis (Fig. 1). With these results and the clinical presentation, a diagnosis of statin-induced autoimmune necrotizing myopathy was confirmed.

**Figure 2.**
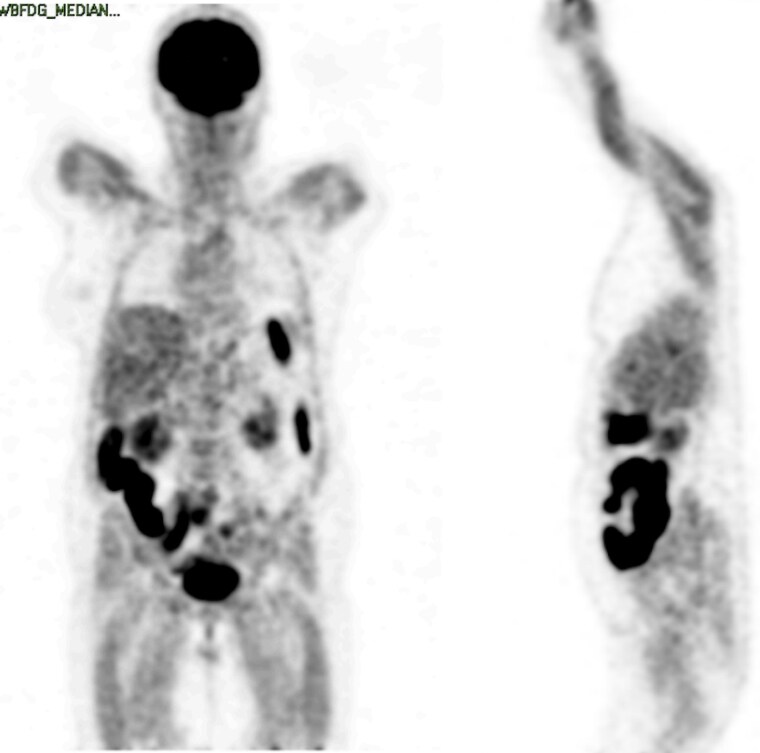
Positron emission tomography computed tomography scan. Positron emission tomography coronal section (A) and sagittal section (B) showing a diffuse increase in radioactive tracer in the muscles of the thoracic and pelvic limb. The distribution of inflammation and the intensity of uptake suggest a generalized inflammatory process, supporting the diagnosis.

A positron emission tomography computed tomography scan showed a diffuse increase in radioactive tracer concentration in the muscles of the thoracic and pelvic limbs, consistent with inflammatory processes (Fig. 2). A dual-energy X-ray absorptiometry scan revealed a 3.7% decrease in muscle mass over a period of 9 months. Over time, the patient regained muscular strength, however, muscle mass did not recover. HLA typing showed the presence of HLA DRB1*08; this has been found to associate with autoimmune disease and antibody production. As part of the differential diagnosis, a molecular panel for muscular dystrophy was conducted, which did not reveal any pathogenic variant of clinical significance.

## Treatment

**Figure 3.**
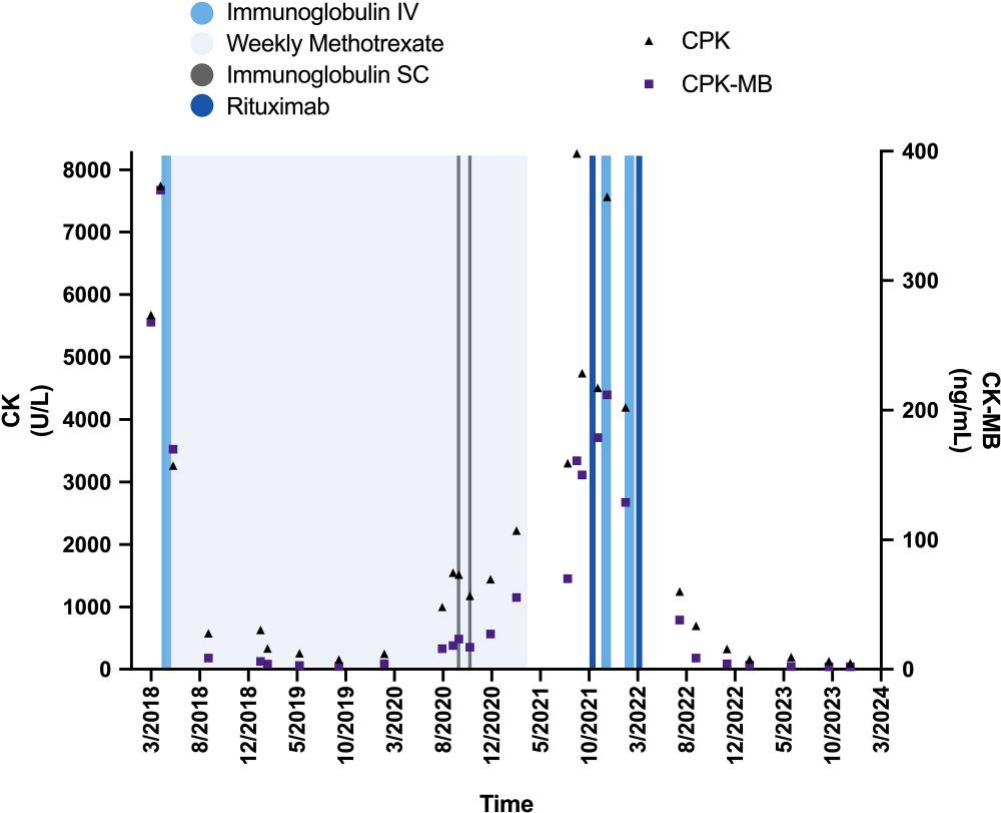
Response to treatment over time. This graph shows the treatment the patient has received over time, along with the concentrations of CK and CK-MB. The induction treatment was a cycle of intravenous immunoglobulin, followed by weekly methotrexate, which controlled the condition for two years. The patient experienced a relapse associated with coronavirus infection, presenting with elevated levels of CK and CK-MB and muscle weakness. Two cycles of intravenous immunoglobulin alternated with 2 cycles of rituximab were administered. This resulted in significant improvement, and the patient has remained in remission to date.

The initial intervention consisted of the immediate and permanent suspension of statin therapy, along with the withdrawal of bezafibrate treatment. Despite these measures, symptoms persisted and CK levels rose to 7749 U/L (129.41 μkat/L). The next step in management was induction therapy with a cycle of intravenous immunoglobulin at a dose of 2 g/kg given over 2 days followed by subcutaneous methotrexate at a dose of 500 mg weekly. This treatment resulted in a marked reduction in CK concentration, which decreased to 3263 U/L (54.49 μkat/L), representing a 58% reduction compared to the previous value. Maintenance therapy with weekly methotrexate was continued for 24 months. The patient experienced a relapse, which coincided with an episode of COVID-19, characterized by an elevation in CK levels. Administration of 2 doses of subcutaneous immunoglobulin failed to elicit improvement. Because of the persistent elevation in CK, 2 cycles of rituximab 500 mg were administered, alternating with 2 cycles of intravenous immunoglobulin at a dose of 2 g/kg. This combined regimen led to complete and sustained remission without recurrence to the present day (28 months). In Fig. 3, the pharmacological management and the corresponding levels of CK and CK-MB are shown.

## Outcome and Follow-up

The patient has been under follow-up since 2018. She has fully recovered muscle strength and independence, with CK and CK-MB levels consistently recorded within normal ranges. Since statin treatment was permanently discontinued, the patient was prescribed evolocumab 140 mg every 2 weeks, which she continued for over a year. With this medication, the patient achieved target levels of low-density lipoprotein (LDL-C) < 70 mg/dL (< 1.81 mmol/L) with no adverse effects. However, because of financial constraints, the patient had to discontinue its use. Currently, she is being treated with ezetimibe 10 mg daily and a very low cholesterol diet (< 200 mg/daily); with this treatment, her LDL-C levels is 109 mg/dL (2.82 mmol/L) (reference range: <100 mg/dL; <2.59 mmol/L), with a target LDL-C level of <70 mg/dL (< 1.81 mmol/L).

## Discussion

HMG-CoA reductase inhibitors, commonly called “statins,” are medications with multiple proven benefits and a safe therapeutic profile. Among the most common complications are myalgia, which may occur in 9% to 20% of statin users. Necrotizing myopathy, another possible complication of statin use, is extremely rare [10]. Given the widespread use of statins as first-line treatment for dyslipidemia worldwide, it is important to be acquainted with this condition and its management.

The mechanism behind anti-HMG-CoA reductase antibody formation is unknown but may involve genetic susceptibility in which class II HLA alleles determine the production of these antibodies. The literature reports such an association primarily with DRB1*11. Autoantibody production is increased upon exposure to statins [11]. In this case, the patient was found to carry the HLA DRB1*08 allele, suggesting that this HLA subtype may also contribute to susceptibility. Evidence of autoimmunity in the patient and her relatives suggests that this could be an influencing factor for this complication.

The patient clinical presentation is in line with other reported cases, beginning with progressive proximal muscle weakness in both upper and lower limbs [12]. The initial laboratory features are characterized by high and sustained CK and CK-MB levels despite statin discontinuation. Elevated lactate dehydrogenase, AST, ALT, and, in rare instances, increased serum creatinine levels may occur because of severe muscle breakdown.

Statin type, dosage, and the duration of exposure varies in patients who develop IMNM. The patient had been on atorvastatin for >5 years at low and intermittent doses. Nevertheless, IMNM has been observed within the first few years of statin use and up to >10 years after use, and occurrence of this condition appears to occur regardless of statin dose or type [1, 3, 5].

Persistent and severe elevation of CK and CK-MB levels after statin discontinuation is an important diagnostic clue. Regenerating muscle fibers express high HMG-CoA reductase levels, which may explain the further CK elevation poststatin withdrawal [10]. In cases of nonnecrotizing myositis, levels decrease upon termination of the drug. The most affected muscle groups are proximal limb muscles, with pronounced weakness but no pain, and in rare instances, dysphagia, arthralgia, myalgia, dark urine, or rash have been reported [5, 12]. In imaging studies, primarily magnetic resonance studies, patients with IMNM have generalized muscle edema, atrophy, and fatty replacement compared to other myopathies [13].

**Figure 4.**
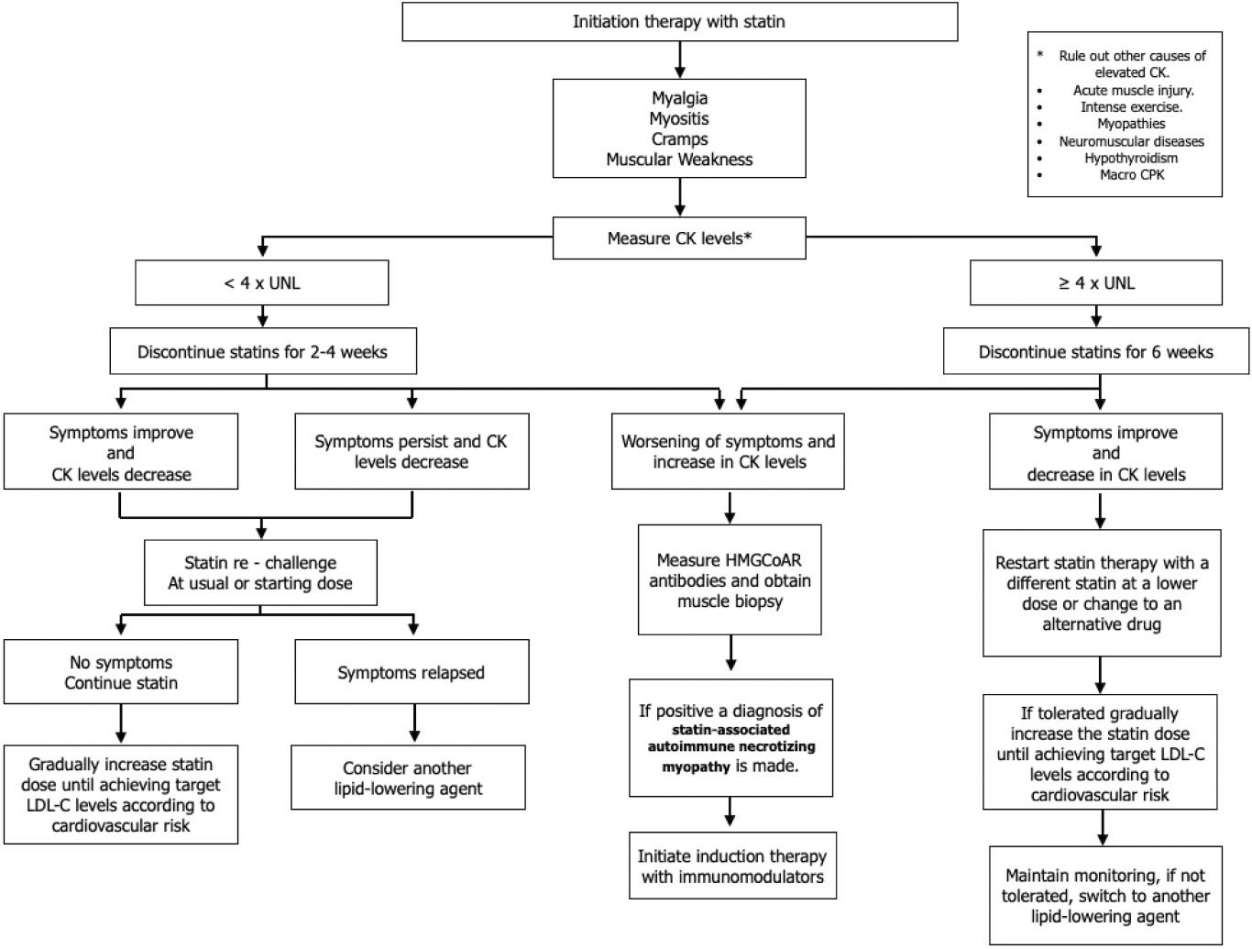
Algorithm for managing adverse effects associated with statin use. Algorithm for the management of patients on statin therapy, including the evaluation of muscle-related side effects and guidance on adjusting or discontinuing treatment based on CK levels and symptoms.

The histological findings in patients with anti-HMG-CoA reductase associated myonecrosis include degeneration of muscle fibers, presence of predominantly M2 macrophages that play a role in muscle regeneration and repair, and CD4+ and CD8+ T cells [14]. In our patient, biopsy findings and the positive HMG-CoA reductase antibody results confirmed the clinical suspicion. Figure 4 presents an algorithm for managing statin-associated muscle adverse effects and the decision-making steps regarding diagnosis and lipid-lowering treatment.

There are no established guidelines or protocols for treating statin-associated myonecrosis. Patients have been managed using diverse treatment regimens. Induction therapies reported in the literature typically involve dual or triple therapy, including intravenous immunoglobulins, methotrexate, with or without glucocorticoids. Maintenance therapy often includes methotrexate, whereas in other cases, azathioprine or rituximab, with or without low-dose steroids may be used [6]. A complete, sustained remission in patients with autoimmune necrotizing myopathy is an infrequent event. This patient responded well to immunoglobulin infusions followed by rituximab. It is important to highlight that relapse in our case, coincided with coronavirus infection; this has been observed to initiate or exacerbate autoimmune phenomena [15]. Regarding lipid-lowering treatment, after an event of this magnitude, another lipid-lowering agent that is not a statin must be administered.

## Learning Points

- Statin-induced immune-mediated necrotizing myopathy is an extremely rare complication but should be suspected in patients who use statins and present with pronounced proximal muscle weakness.
- It primarily affects the proximal muscles of the limbs, leading to pronounced weakness with no pain. In rare cases, additional symptoms such as dysphagia, arthralgia, myalgia, dark urine, or rash may also be reported.
- Diagnosis is established by persistent elevation of CK and CK-MB despite discontinuation of the drug. The diagnosis is confirmed by the presence of myonecrosis in a biopsy of an affected muscle and positive titers of anti-HMG-CoA reductase antibodies.
- The therapeutic options reported in the literature typically include both induction and maintenance therapies. In our case, the most effective treatment was a combination of intravenous immunoglobulin followed by rituximab, with which, complete remission has been achieved.

## Data Availability

Original data generated and analyzed during this study are included in this published article.
